# Mice lacking two alleles of the schizophrenia risk gene *Tcf4* and *Olig2* display deficits in anxiety-related behavior, sensorimotor gating, and cognition

**DOI:** 10.3389/fncel.2026.1837159

**Published:** 2026-07-01

**Authors:** Man-Hsin Chang, Ruofan Li, Marius Stephan, Andrea Schmitt, Peter Falkai, Moritz J. Rossner

**Affiliations:** 1 Department of Psychiatry and Psychotherapy, Molecular and Behavioral Neurobiology, LMU University Hospital, LMU Munich, Munich, Germany; 2 International Max Planck Research School for Translational Psychiatry (IMPRS-TP), Munich, Germany; 3 Systasy Bioscience GmbH, Munich, Germany; 4 Laboratory of Neuroscience (LIM27), Institute of Psychiatry, University of São Paulo, São Paulo, Brazil; 5 Department of Clinical Translation, Max Planck Institute of Psychiatry, Munich, Germany; 6 German Center for Mental Health (DZPG), Partner Site Munich/Augsburg, Munich, Germany

**Keywords:** mental disorders, mouse behaviors, myelination, *Olig2*, oligodendrocytes, *TCF4*

## Abstract

**Introduction:**

Oligodendrocytes (OLs) and myelination contribute to higher-order cognitive functions, and are impaired in several mental disorders, including schizophrenia (SZ). The SZ risk gene *transcription factor 4* (*TCF4*) and *OLIG2* are two critical regulators of differentiation and maturation of oligodendrocyte precursor cells (OPCs) and OLs. *Tcf4*-*Olig2* double heterozygous deletion (*Tcf4*-*Olig2* dHet) mice have been shown to exhibit impairments in OL differentiation and myelination.

**Methods:**

Therefore, we performed a comprehensive behavioral profiling using a Research Domain Criterion (RDoC)-based pipeline in this model.

**Results:**

*Tcf4*-*Olig2* dHet mice showed impaired place learning in the positive valence system, reduced anxiety-like behaviors in the negative valence system, impaired remote fear memory in the cognitive domain, and abnormal sensorimotor gating.

**Discussion:**

Thus, subtle OLs and myelination impairments in *Tcf4*-*Olig2* dHet mice led to several psychiatric disorder-related behavioral deficits. We conclude that *Tcf4*-*Olig2* dHet mice represent a preclinical mouse model to study myelination-related mechanisms relevant for mental disorders.

## Introduction

Oligodendrocytes (OLs) and the formation of myelin sheaths are increasingly recognized as crucial contributors to higher-order brain functions, including learning, memory, and cognitive flexibility (Fields, 2008). Myelination not only facilitates rapid and efficient transmission of action potentials along the axons (Bradl and Lassmann, 2010) but also regulates the development and plasticity of neuronal networks (Takahashi et al., 2011). Research of cognitive dysfunction and pathological behaviors in psychiatric disorders has been so far dominated by neuron-centric hypotheses, focusing particularly on synaptic plasticity and neurotransmitter imbalance (Petrik et al., 2012; Howes and Onwordi, 2023). However, accumulating evidence from genetic, imaging, and postmortem studies suggests that abnormal white matter integrity is not merely a secondary feature but possibly also a primary driver of multiple psychiatric disorders, including SZ, autism spectrum disorder, and attention deficit hyperactivity disorder (Fields, 2008; Raabe et al., 2019). These findings imply that OL/myelination dysfunction likely plays a critical role in the patho-mechanisms underlying cognitive and behavioral impairments. Despite these associations, it remains unclear whether OL/myelination deficits are causal contributors to psychiatric disorders or secondary consequences of altered neuronal activity. To address this question, an animal model with selective OL/myelination disruption is needed for assessing the roles of OL/myelination in behavioral phenotypes relevant to psychiatric disorders.

*Transcription factor 4* (*TCF4*) gene encodes a basic helix–loop–helix (bHLH) protein that regulates the expression of multiple downstream target genes and the differentiation of neuronal and glial progenitor cells during central nervous system development (Quednow et al., 2014). Genome-wide association studies (GWAS) have provided strong evidence for *TCF4* to be linked with several psychiatric disorders, including SZ, bipolar disorder, and post-traumatic stress disorder (Del-Favero et al., 2002; Ripke et al., 2011; Steinberg et al., 2011; Gelernter et al., 2019), underscoring its importance in neurodevelopmental and higher-order brain function (Quednow et al., 2014). In addition, *Tcf4* transgenic mice have been revealed to exhibit deficits in cognitive and sensorimotor gating functions (Brzózka et al., 2010; Brzózka and Rossner, 2013; Stephan et al., 2022). Similarly, the *OLIG2* gene encodes a bHLH transcription factor that serve as a master regulator of OL specification, differentiation, and maturation (Novitch et al., 2001). Although *OLIG2* is not among the top GWAS hits, there are reports that link *OLIG2* and its interactors with SZ and psychosis (Georgieva et al., 2006; Sims et al., 2009; Falkai et al., 2023). TCF4 is the obligate hetero-dimerization partner of OLIG2 and both factors are expressed in OLs (Wedel et al., 2020). Previous work using a mouse model with heterozygous deletion of both *Tcf4* and *Olig2* demonstrated that these transcription factors cooperate to regulate OL differentiation and myelination in a cell-autonomous manner (Wedel et al., 2020). *Tcf4*-*Olig2* double heterozygous deletion (*Tcf4*-*Olig2* dHet) mice therefore provide a genetic model to investigate the functional consequences of impaired OL development with increased validity for mental disorders.

However, the behavioral phenotypes of *Tcf4-Olig2* dHet mice have not yet been characterized. In the present study, we aim to bridge this gap by conducting a comprehensive behavioral phenotyping of *Tcf4-Olig2* dHet mice using a Research Domain Criterion (RDoC)-based PsyCoP pipeline (Volkmann et al., 2021). RDoC, developed by the National Institute of Mental Health (NIMH), is a translational research framework that shifts the focus from traditional diagnostic categories toward functional dimensions of behavior and cognitive/emotional processes. RDoC enhances the translational potential of animal research for addressing neurobiological mechanisms underlying psychopathology rather than modeling specific psychiatric disorders (Cuthbert, 2020). Building upon this framework, we developed the PsyCoP (Systematic Behavioral and Cognitive Profiling) platform for mice, which systematically implements the RDoC framework in behavioral phenotyping analyses (Volkmann et al., 2021; Stephan et al., 2022). Centered on the RDoC domains, the PsyCoP platform establishes a systematic behavioral testing pipeline that enables multidimensional behavioral assessment of animal models. Here we use this approach (Volkmann et al., 2021) to determine if OL/myelination deficits can recapitulate the behavioral endophenotypes observed in human psychiatric disorders, thereby establishing a novel model for studying the OL/myelination hypothesis of mental illness. Furthermore, sex-stratified mouse behaviors would be evaluated due to the fact that several mental disorders exhibit sex differences in the incidence, symptomatology, and responses to treatment (Gobinath et al., 2017; Riecher-Rössler, 2017).

## Materials and methods

### Animals

All animal experiments followed the institutional animal care and complied with the committee guidelines of the LMU University Hospital, LMU Munich. Tcf4-4-fl^fl/0^Olig2-Cre^tg/0^ (*Tcf4*-*Olig2* dHet) mice were derived from crossing Tcf4-4-fl^fl/fl^ and Olig2-Cre^tg/0^ mice, which were maintained on a C57Bl/6 N background. The littermate Tcf4-4-fl^fl/0^Olig2-Cre^+/+^ mice were used as wild type (WT) controls. 4 to 6 animals were housed together in a holding cage and up to 17 animals were housed together in the IntelliCage with the same sex and balanced genotypes under a 12:12 h (hr) light/dark cycle, and provided with enrichment and food *ad libitum*. Water was conditionally restricted only in the IntelliCage System. Experimental animals of both sexes were 8–17 weeks old at the time of testing. 129/Sv female mice with oophorectomy were used as social stimulus mice in the social interaction test.

### Behavioral tests

**Figure 1:**
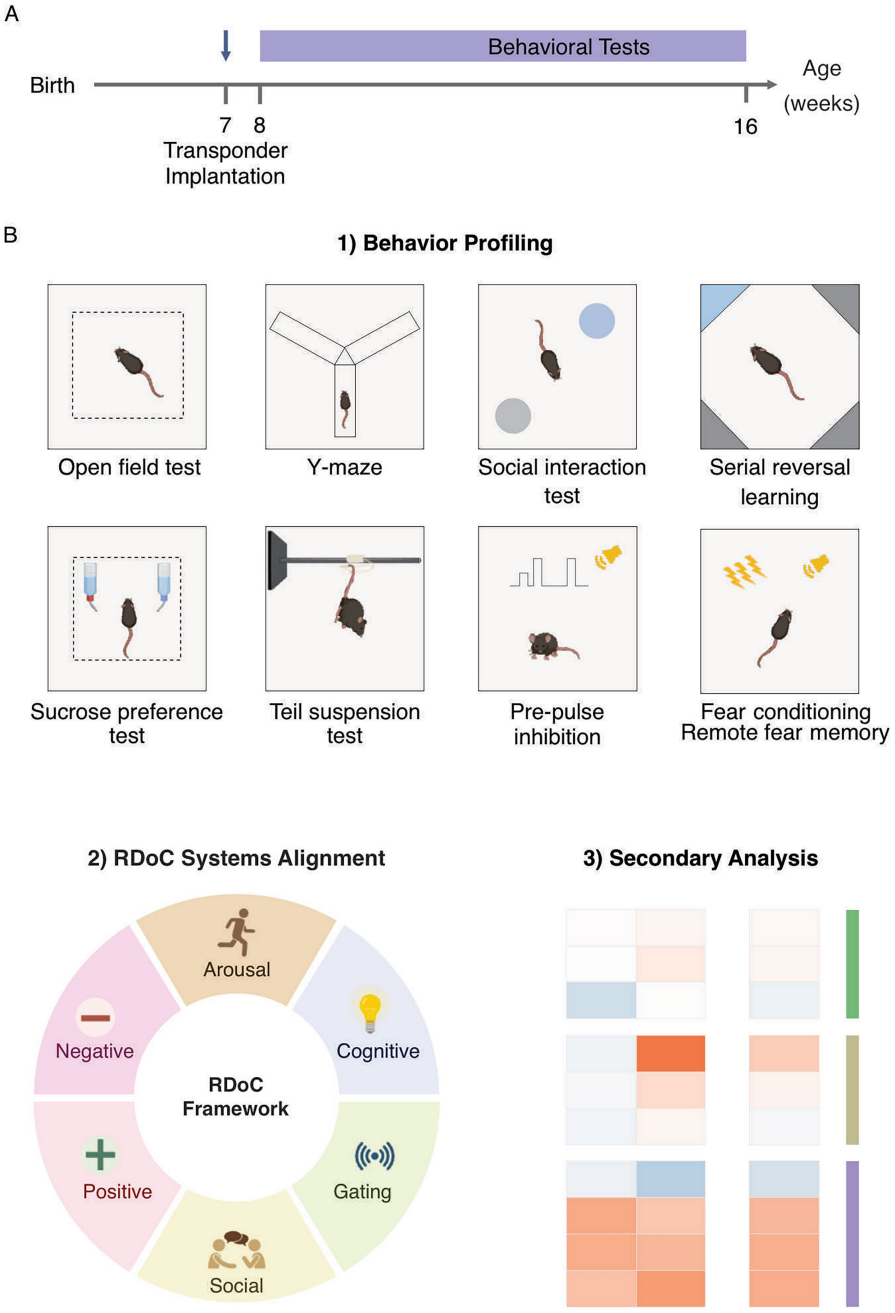
Schematic workflow of behavioral phenotyping. **(A)** The timeline of behavioral tests. **(B)** Overview workflow of behavioral profiling, RDoC systems alignment, and secondary analysis.

One week before the behavioral tests, mice were implanted with radio frequency identification (RFID) chips for recognition (Figure 1A). At least 10 min (min) before each testing session, the animals were transported in their home cages to the experiment room for habituation. To minimize the stress induced by the traditional tail handling, experimental mice were taken out from their cages using a transparent polycarbonate tunnel (Hurst and West, 2010). All equipment was cleaned between tests using sodium dodecyl sulfate solution followed by ethanol to remove olfactory cues. All mouse experiments were recorded and analyzed by ANY-maze software (Stoelting, Wood Dale, IL, USA) unless otherwise stated. All behavioral tests were conducted as previously described (Volkmann et al., 2021), and the detailed procedures can be found in the Supplementary material.

### Statistics

All data are presented as mean ± standard deviation (SD). The n values indicate the number of individual subjects analyzed per group. Statistical analyses were performed in R (version 4.2.3), the analysis script is available on: https://github.com/shabear22/Mouse-Behavioral-Test. Data normality was assessed using the Shapiro–Wilk tests, and homogeneity of variances were evaluated with Levene’s test. Data were then examined with a two-way ANOVA or a linear mixed-effects model for repeated measurement and corrected for multiple comparison using Benjamini-Hochberg false discovery rate (FDR) test. The datasets with significant interaction were further analyzed with an estimated marginal means *post hoc* test. Pooled data with both sexes were analyzed using either t test or Mann–Whitney U test. Z-scores were calculated by standardizing each raw value (X) to the population mean (*μ*) and standard deviation (*σ*) with the following formula:


$$Z=\frac{X-\mu \;}{\sigma }$$

Values from the control group were used as the reference for calculating and visualizing the distance of two clusters in the heatmaps. Statistical significance was set as *p < 0.05; **p < 0.01; and ***p < 0.001.

## Results

*Tcf4-Olig2* double heterozygous deletion (*Tcf4-Olig2* dHet) mice display OL differentiation and myelination deficits (Wedel et al., 2020). To study whether the impairments in OLs/myelination lead to behavioral alterations, a series of behavioral tests using the PsyCoP battery (Volkmann et al., 2021) was conducted (Figure 1B).

### *Tcf4-Olig2* dHet show impaired place learning performance in the positive valence and decreased anxiety-like behaviors in the negative valence systems

**Figure 2:**
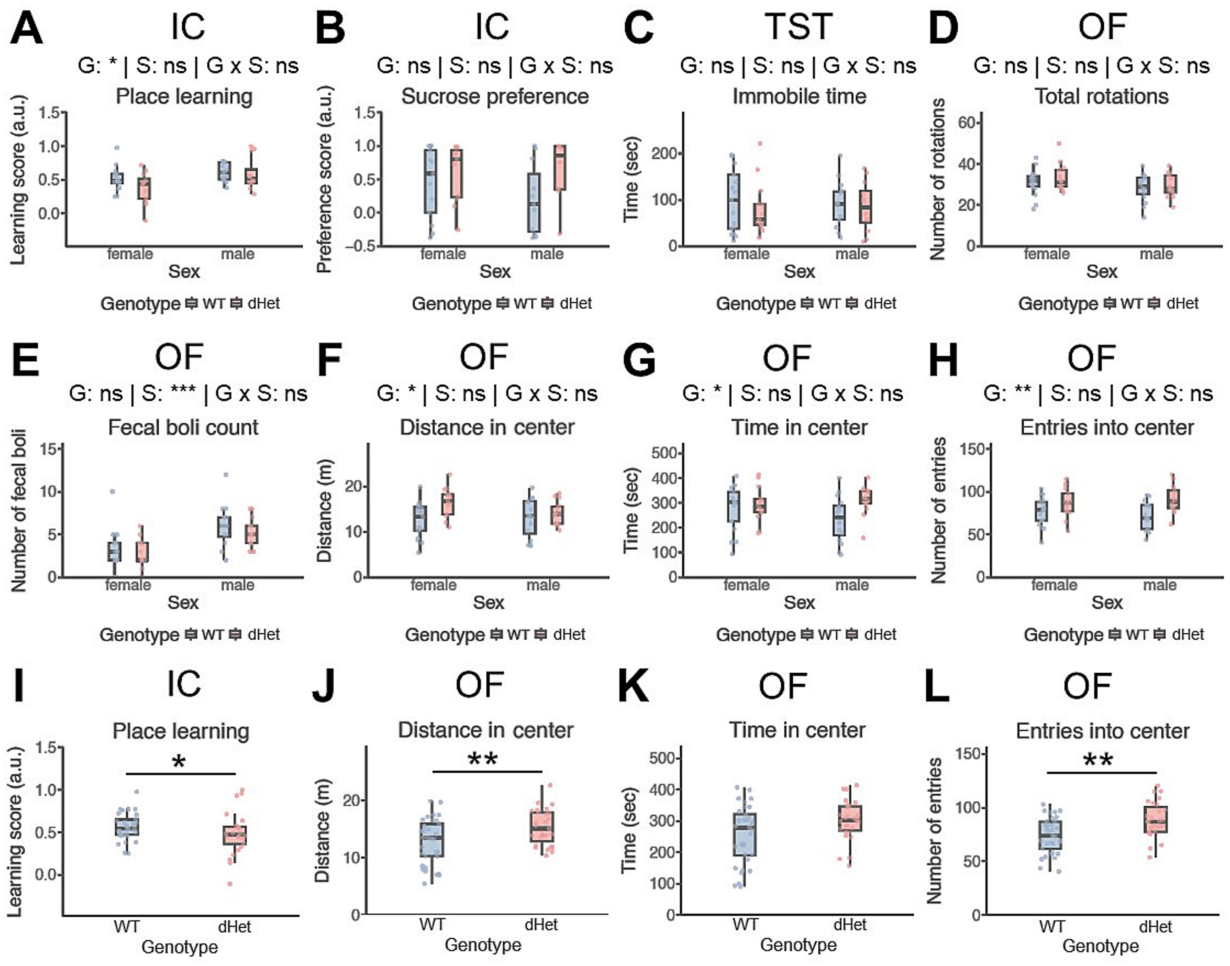
*Tcf4-Olig2* dHet mice showed declined place learning performance and lower anxiety-like behaviors in positive and negative valence systems. **(A)** The learning performance in the place learning paradigm and **(B)** the preference score in the sucrose preference test in the IntelliCage (IC). **(C)** The time of immobility in the tail suspension test (TST). **(D)** The number of rotations and **(E)** fecal pellets in the open field (OF) test. **(F)** The total traveled distance, **(G)** time spent, and **(H)** the number of entries into the central area of the OF. Two-way ANOVA test: *p < 0.05; **p < 0.01; ***p < 0.001. G, genotype effect; S, sex effect; G × S, genotype × sex interaction. **(I)** The learning performance of pooled sexes in the place learning. **(J)** The total traveled distance, **(K)** time spent, and **(L)** the number of entries of pooled sexes into the central area of the OF. *t*-test for **(J,L)**; Mann–Whitney *U*-test for **(I,K)**: *p < 0.05; **p < 0.01. Data = Mean ± SD. WT: *n* = 33 (16 males and 17 females), dHet: *n* = 27 (14 males and 13 females).

Positively-reinforced spatial learning was assessed by a preference score reflecting the learning performance. A two-way ANOVA analysis revealed a significant effect of genotype but neither sex nor genotype x sex interaction showed a significant effect (Figure 2A; *p* = 0.014 for genotype effect), and *Tcf4-Olig2* dHet with pooled sexes showed lower learning ability (Figure 2I, 0.34 ± 0.48 vs. 0.56 ± 0.16, *p* = 0.0181). Anhedonia-like behavior was assessed by the sucrose preference test, and no differences were found between groups (Figure 2B). These data indicate that *Tcf4-Olig2* dHet with both sexes showed declined learning performance.

To assess depressive-like behavior in *Tcf4-Olig2* dHet mice, the tail suspension test was conducted. The higher the immobility time and episode, the more depressive-like the mice are (Cryan et al., 2005). *Tcf4-Olig2* dHet mice showed no significant differences in the total time of immobility (Figure 2C) compared to the control mice. These data indicate that *Tcf4-Olig2* dHet mice manifested no depressive-like phenotypes.

The explorative activity and anxiety levels of *Tcf4-Olig2* dHet mice were investigated in the open field (OF) test. The frequency of directional changes or rotations, while ambulating inside the OF box, was used as an indicator of novelty- or anxiety-induced hyperactivity. *Tcf4-Olig2* dHet mice showed no alterations in total rotations (Figure 2D). The number of fecal pellets and the duration staying in the center of the OF is thought to reflect the anxiety level, as mice produced more fecal boli and spent less time in the center when being more anxious (Seibenhener and Wooten, 2015). There were no effects of genotype and genotype x sex interaction in the fecal pellet number, while a significant effect of sex was revealed (Figure 2E; *p* = 0.0000256 for sex effect). However, ANOVA analyses showed significant effects of genotype in center distance (Figure 2F; *p* = 0.01), center time (Figure 2G; *p* = 0.032), and center entries (Figure 2H; *p* = 0.001). *Tcf4-Olig2* dHet mice with pooled sexes traveled a significantly longer distance (Figure 2J, 15.28 ± 3.12 vs. 12.83 ± 3.98, *p* = 0.0097), spent a longer time (Figure 2K, 301.2 ± 64.27 vs. 256.08 ± 91.67, *p* = 0.074), and also made significantly more entries into the center area compared to the control (Figure 2L, 88.63 ± 16.46 vs. 74.06 ± 16.47, *p* = 0.0012). These data suggest that *Tcf4-Olig2* dHet mice with both sexes were less anxious.

### *Tcf4-Olig2* dHet show no alterations in the social valence

To investigate the social interaction function of *Tcf4-Olig2* dHet mice, social preference and social memory tests were performed. The social preference index was determined and showed no differences between groups (Supplementary Figure S1A). The social discrimination index was calculated to assess social memory, while no significant differences were revealed (Supplementary Figure S1B). These findings indicate that social interaction was intact in *Tcf4-Olig2* dHet mice.

### *Tcf4-Olig2* dHet show subtle learning deficits in the cognitive system and irregular sensorimotor gating function in the gating system

To study the spatial working memory in *Tcf4-Olig2* dHet mice, Y maze test was conducted. The spontaneous alteration is an indicator of the spatial working memory, mice with intact memory function remember the previously-explored arm and tend to enter a new arm, as mice have a tendency to explore novel regions (Kraeuter et al., 2019). *Tcf4-Olig2* dHet mice showed no differences in the spontaneous alterations (Supplementary Figure S2A). These results suggest that the working memory of *Tcf4-Olig2* dHet mice is normal.

A serial reversal learning paradigm in the IntelliCage system was conducted by changing the assigned drinking corner every day during the test to examine the learning flexibility of the mice. There were no significant effects of genotype and genotype x sex interaction, while sex exhibited a significant effect in serial reversal learning (Supplementary Figures S2B–S2D, *p* = 0.00000194 for sex effect).

**Figure 3:**
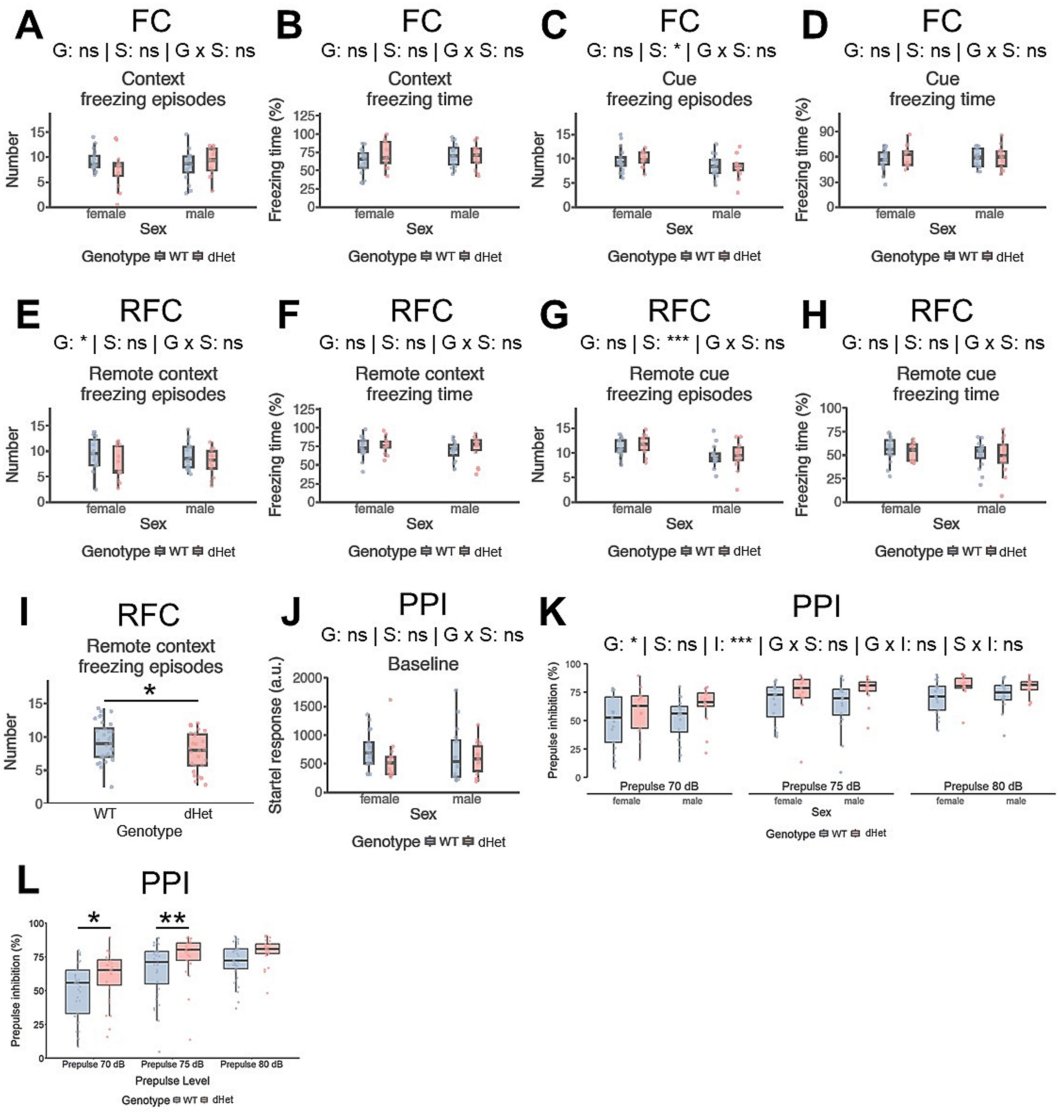
*Tcf4-Olig2* dHet mice showed impaired remote fear memory and abnormal prepulse inhibition in cognitive and sensorimotor gating systems. **(A)** The freezing episodes, **(B)** the freezing time during context stage, **(C)** the freezing episodes, and **(D)** the freezing time during cue stage in the fear conditioning test (FC). **(E)** The freezing episodes, **(F)** the freezing time during context stage **(G)** the freezing episodes, and **(H)** the freezing time during cue stage in the remote fear memory test (RFC). Two-way ANOVA test: *p < 0.05; ***p < 0.001. G, genotype effect; S, sex effect; G × S, genotype × sex interaction. **(I)** The freezing episodes of pooled sex during context stage in the RFC. *t*-test: *p < 0.05. **(J)** The baseline of startle response in the prepulse inhibition (PPI) test. **(K)** The percentage of PPI with 70 dB, 75 dB, and 80 dB prepulse. Linear mixed-effects model: *p < 0.05; ***p < 0.001. G, genotype effect; S, sex effect; I, intensity effect; G × S, genotype × sex interaction; G × I, genotype × intensity interaction; S × I, sex × intensity effect. **(L)** The percentage of PPI of pooled sexes with 70 dB, 75 dB, and 80 dB prepulse. Linear mixed-effects with estimated marginal means *post hoc* test: *p < 0.05; **p < 0.01. Data = Mean ± SD. WT: *n* = 33 (16 males and 17 females), dHet: *n* = 27 (14 males and 13 females).

To study the associative learning and memory of *Tcf4-Olig2* dHet mice, a fear conditioning test was conducted. Neither genotype nor genotype x sex interaction showed significant effects in both freezing episodes and time during context (Figures 3A,B) and cue (Figures 3C,D) stages. While a significant effect of sex was revealed in the freezing episodes during cue stage (Figure 3C; *p* = 0.018), suggesting a different response between male and female mice to this parameter. OLs and myelination are known to play key roles in learning and memory, especially during the consolidation of short-term memory from the hippocampus to the long-term memory stored in the prefrontal cortex (Fields and Bukalo, 2020). To examine the long-term memory, a remote fear memory test was conducted about 20 days after the fear conditioning test. A significant effect of genotype in the freezing episodes during context stage was found (Figure 3E; *p* = 0.043), and *Tcf4-Olig2* dHet mice with pooled sexes showed reduced freezing number compared to the control (Figure 3I; 7.71 ± 2.86 vs. 9.28 ± 2.88, *p* = 0.04). There were no differences in the freezing time during context stage (Figure 3F), and in the freezing episodes (Figure 3G) and time during cue stage (Figure 3H). A significant effect of sex was revealed in the freezing episodes during cue stage (Figure 3G; *p* = 0.0009). These findings suggest that the function of forming recent memory was intact, while the remote fear memory was impaired in *Tcf4-Olig2* dHet mice.

To study the sensorimotor gating function, which is commonly impaired in SZ and several other psychiatric disorders (Geyer, 2006), a prepulse inhibition (PPI) test with different levels of prepulses was performed. There were no differences in the baseline startle responses between *Tcf4-Olig2* dHet and the control mice (Figure 3J). Significant effects of genotype and intensity in the prepulse inhibition were revealed (Figure 3K; *p* = 0.0215 for genotype effect; *p* = 3.706E-18 for intensity effect). *Tcf4-Olig2* dHet mice with pooled sexes showed significantly elevated PPI at 70 dB (Figure 3L; 60.67 ± 18.75 vs. 50.38 ± 21, *p* = 0.0291), and 75 dB prepulse (Figure 3L; 75.6 ± 16.07 vs. 63.04 ± 23.18, *p* = 0.0082). These data showed that *Tcf4-Olig2* dHet mice had higher PPI, indicating an abnormally more sensitive sensorimotor gating function.

### Female mice showed elevated nocturnality in the arousal system

Ambulation in the OF measures the response to a novel environment as the mean speed, i.e., the total distance traveled over the time of the experiment. *Tcf4-Olig2* dHet mice showed no alterations in the mean speed (Supplementary Figure S3A). These data suggest that the novelty-induced locomotion was not altered in *Tcf4-Olig2* dHet mice.

To separate novelty-induced from general activity, the overall activity in the IntelliCage was continuously monitored over several days. The general activity in the home cage showed no differences between *Tcf4-Olig2* dHet and the control mice (Supplementary Figure S3B). The circadian activity was assessed by calculating a nocturnality score, i.e., the preference for nighttime over daytime activity. Neither genotype nor genotype x sex interaction showed significant effects, while a significant effect from sex was revealed in the nocturnality (Supplementary Figure S3C; *p* = 0.0002 for sex effect).

### Z-scores of individual behavioral tests revealed moderate to strong phenotypes in the negative, positive, cognitive, and sensorimotor gating domains

**Figure 4:**
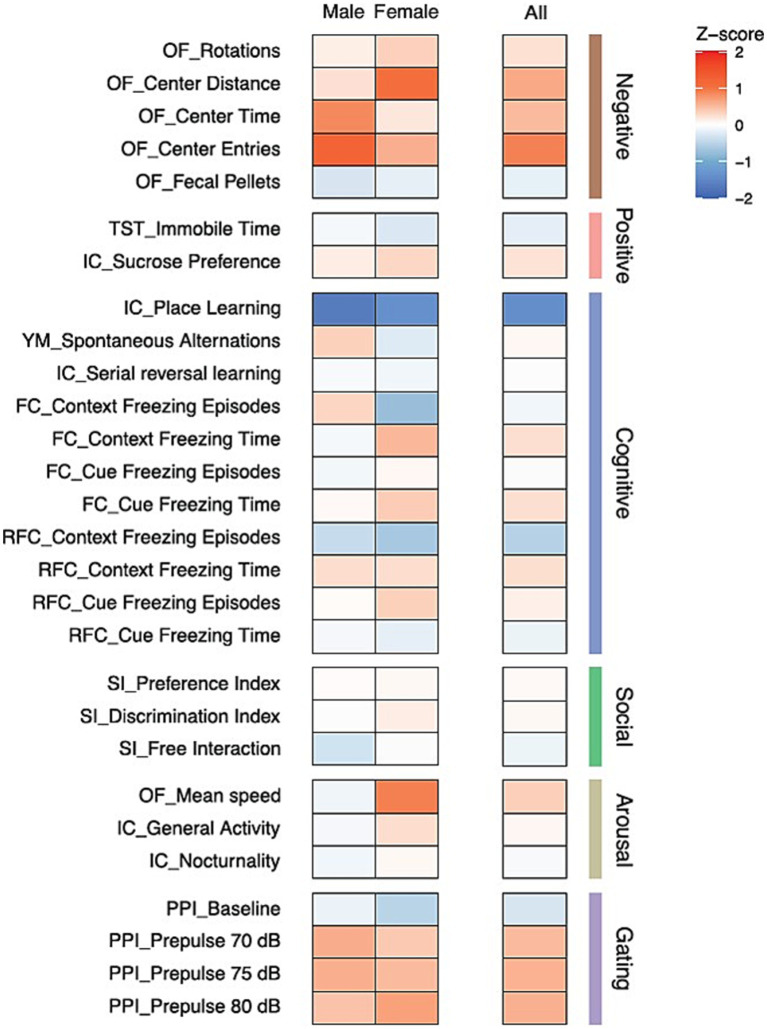
Z-scores of all behavioral tests groped by RDoC revealed dysfunction in negative, positive, cognitive, and gating systems. The heatmap illustrates changes relative to the WT group. (Left panel) Z-scores of individual behavioral test with sex stratification into male and female mice. (Right panel) Z-scores of individual behavioral test.

To reveal the behavioral patterns across different valences under RDoC, Z-scores of each behavioral tests were determined with the control group as the reference. The color scale representing Z-scores demonstrates the distance between clustered values of *Tcf4-Olig2* dHet mice from the values of WT mice, reflecting the differences of behavioral phenotypes between groups. The phenotype association of the Z-score directionality with respect to its interpretation as a potential pathological feature can be found in the Supplementary Table S3. *Tcf4-Olig2* dHet mice exhibited strong phenotypes in the negative, positive, cognitive, and sensorimotor gating systems comparing to WT mice (Figure 4, right panel). The effects of sexes were also determined, with female *Tcf4-Olig2* dHet mice showing larger variances in behavioral patterns across negative, cognitive, arousal, and sensorimotor gating systems, while male *Tcf4-Olig2* dHet mice showing alterations mainly in the negative, cognitive, and sensorimotor gating systems comparing to WT mice (Figure 4, left panel). It needs to be noted that not all visible differences are significant in the statistical tests applied. Therefore, the heatmap should be regarded as a descriptive “soft” visualization tool suitable for easy and intuitive access of the comprehensive behavioral data of this study. Taken together, *Tcf4-Olig2* dHet mice demonstrated phenotypes in the negative, positive, cognitive, and gating functions.

## Discussion

*Tcf4-Olig2* dHet mice were shown to have impaired OL differentiation (Wedel et al., 2020). To study if they manifest any psychiatric disorders-relevant behavioral phenotypes, we performed a series of behavioral tests and employed the RDoC-based PsyCoP platform to systematically integrate multi-task behavioral measures (Volkmann et al., 2021). Through standardized z-score normalization and heatmap-based visualization, the platform enables researchers to evaluate behavioral alterations in an intuitive, condensed, and systematic manner.

In the positive valence system, *Tcf4-Olig2* dHet mice exhibited decreased scores in the positively-reinforced place learning in the IntelliCage system (Figures 2A,I). This suggests a modest impairment in learning performance, which is consistent with the knowledge that OLs are critical for motor skill learning and spatial working memory. A previous study showed comparable findings. *Myelin regulatory factor* (*Myrf*) encodes a key transcription factor for OL differentiation. *Myrf* null mutant mice showed significantly lower performance in the maze test compared to control mice, indicating a deficit in the spatial working memory (Shimizu et al., 2023).

In the negative valence system, thigmotaxis was examined. Thigmotaxis describes that mice tend to stay close to the walls rather than explore the center open area, an increase of this behavior is interpreted as an anxiety-like response (Simon et al., 1994). *Tcf4-Olig2* dHet mice showed increased traveled distance, time, and entries in the center, indicating lower anxiety levels. Previous studies using other OLs/myelination-impaired mouse models also showed similar results, including a dietary cuprizone model (Xu et al., 2009). *Plp1* encodes the major myelin protein PLP, and *Plp1* null mutants showed declined anxiety-like behaviors (Tanaka et al., 2009). *Cnp1* encodes another important myelin protein CNP, and similar phenotypes were observed in *Cnp1* null mutant mice (Edgar et al., 2011).

In the cognitive valence system, *Tcf4-Olig2* dHet mice showed no changes in the recent fear conditioning test (Figures 3A–D) but impaired remote fear memory (Figures 3E,I). This finding corresponds to a previous study also showing that myelination deficits contributed to only remote, but not recent, fear memory impairment in a conditional *Myrf* null mutant mouse line (Pan et al., 2020). *Tcf4-Olig2* dHet displayed remote contextual fear memory deficits only by measuring reduced freezing episodes and the effect disappeared with sex stratification and thus represents a subtle effect. This phenotype may be enhanced by adding a second hit, e.g., environmental factor, to the model. Moreover, the number of animals per sex in such a follow-up study should be higher to increase the power to strengthen the detection of subtle to moderate behavioral deficits.

Remote memory consolidation from the hippocampus to the cortex requires OPC differentiation to OL and new myelination formation over time, and the retrieval of remote fear memory also depends on myelination of the underlying circuitry (Fields and Bukalo, 2020). Thus, the deficits of OLs in *Tcf4-Olig2* dHet mice may likely disrupt myelination-dependent remotely connected network assemblies, leading to impaired long-term fear memory formation and retrieval.

In the gating system, *Tcf4-Olig2* dHet mice exhibited abnormally increased PPI (Figures 3K,L), which is different from the decreased PPI found in most studies of SZ patients (Mena et al., 2016) and SZ-relevant mouse models (Featherstone et al., 2007; Bitanihirwe et al., 2010). Several mouse models with OLs/myelination deficits, including *NRG1* mutant, *Nogo-A* mutant, *MBP* mutant, and cuprizone-treated mice, showed reduced PPI (Poggi et al., 2016; Gouvêa-Junqueira et al., 2020). Nonetheless, increased PPI was found in animals under physical stress, which was linked to a disrupted DA system (Pijlman et al., 2003). The abnormal PPI of the startle response in *Tcf4-Olig2* dHet mice indicates alterations in the underlying neuronal circuitry, which has been shown to depend on properly connected cortico-striato-pallido-pontine (CSPP) circuitry (Quednow et al., 2018). Moreover, at the cellular level, parvalbumin-positive interneurons are likely to be involved in PPI modulation in various psychiatric conditions (Isaacs and Riordan, 2020) and have recently been shown to be myelinated (Benamer et al., 2020). It may thus be possible, that not only myelination-dependent remote but also local cortical circuitry may be altered in *Tcf4-Olig2* dHet mice. However, the clinical analog of this abnormal gating function is still unclear, future studies are definitively needed to further evaluate these aspects.

Given the genetic association of *TCF4* with several mental disorders and the moderate reduction in gene dosage in oligodendrocyte-lineage cells*, Tcf4-Olig2* dHet likely represent a mouse model of increased construct validity compared to homozygous deletion models of other regulatory and structural myelin genes. Nonetheless, convergent features (cognitive/remote memory deficits) as well as divergent features (elevated rather than reduced PPI, reduced rather than increased anxiety-like behavior) show myelination-dependent circuit alterations but limited face validity for SZ. Some of the findings presented were modulated by sex, which corresponds to previous findings that rodents exhibit different behavioral phenotypes between sexes (An et al., 2011; Börchers et al., 2022). Most mental disorders have a genetic component but are also influenced by environmental stress. Therefore, *Tcf4*-*Olig2* dHet should be paired in the future with a relevant environmental stressor, such as social defeat and/or social isolation, to generate a “2-hit” mouse model in which eventually more disease-associated phenotypes will be precipitated or enhanced. According to the study from Wedel et al., *Tcf4*-*Olig2* dHet mice do only display oligodendroglial differentiation defects until early postnatal stages and it remains to be determined if functional deficits in oligodendrocyte-lineage cells or myelin plasticity persist at later stages of brain development and affect network maturation and behavior.

Taken together, *Tcf4*-*Olig2* dHet likely represent a preclinical mouse model with increased construct validity to study aspects of myelination-related mechanisms relevant for mental disorders.

## Data Availability

The datasets presented in this study can be found in online repositories. The names of the repository/repositories and accession number(s) can be found at: https://github.com/shabear22/Mouse-Behavioral-Test.
