# Supplementary figures and images for: Mice lacking two alleles of the schizophrenia risk gene Tcf4 and Olig2 display deficits in anxiety-related behavior, sensorimotor gating, and cognition
Source: Front Cell Neurosci. 2026 Jul 1;20:1837159. doi: 10.3389/fncel.2026.1837159 (PMC13368340; doi:10.3389/fncel.2026.1837159)

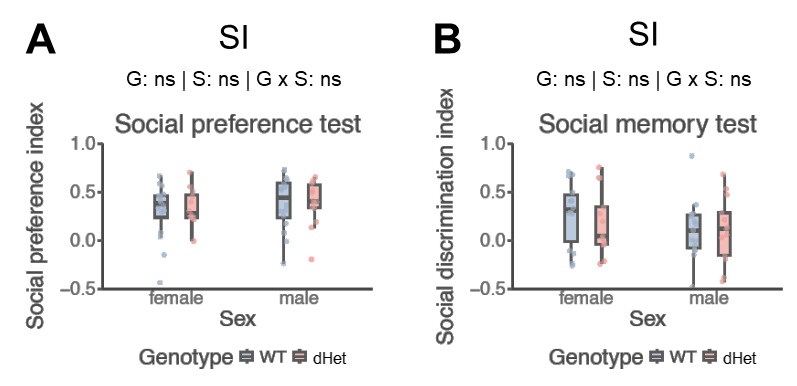

Supplement: Supplementary file 1 [file Image_1.JPEG]

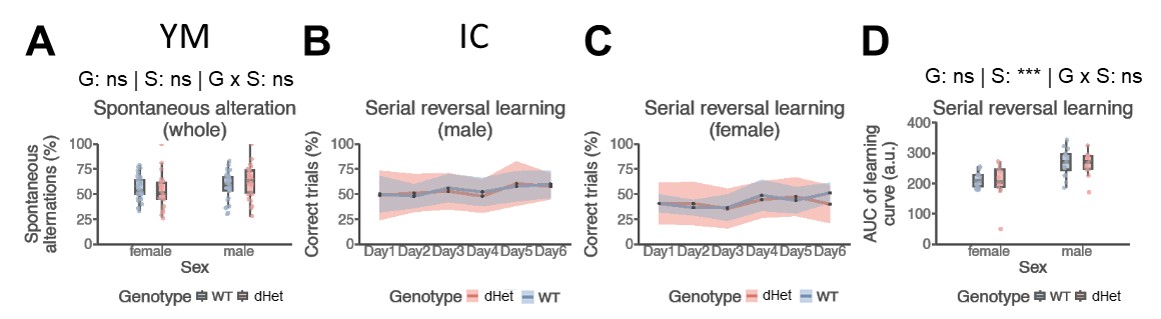

Supplement: Supplementary file 2 [file Image_2.JPEG]

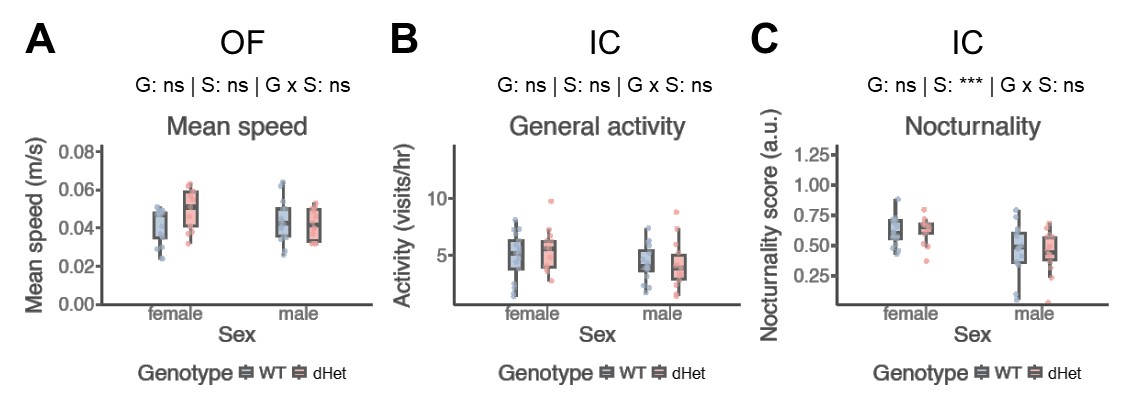

Supplement: Supplementary file 3 [file Image_3.JPEG]
