# Supplementary material for: Mice lacking two alleles of the schizophrenia risk gene Tcf4 and Olig2 display deficits in anxiety-related behavior, sensorimotor gating, and cognition
Source: Front Cell Neurosci. 2026 Jul 1;20:1837159. doi: 10.3389/fncel.2026.1837159 (PMC13368340; doi:10.3389/fncel.2026.1837159)
Supplement: Supplementary file 4 [file Supplementary_file_1.docx]

**Supplementary materials**

**Mice lacking two alleles of the schizophrenia risk gene *Tcf4* and *Olig2* display deficits in anxiety-related behavior, sensorimotor gating, and cognition**

**Man-Hsin Chang^1,2#^, Ruofan Li^1#^, Marius Stephan^1,3^, Andrea Schmitt^1,4^, Peter Falkai^1,5,6^ Moritz J. Rossner^1*^**

^1^Department of Psychiatry and Psychotherapy, Molecular and Behavioral Neurobiology, LMU University Hospital, LMU Munich, 80336 Munich, Germany

^2^International Max Planck Research School for Translational Psychiatry (IMPRS-TP), 80804 Munich, Germany

^3^Systasy Bioscience GmbH, 81669 Munich, Germany

^4^Laboratory of Neuroscience (LIM27), Institute of Psychiatry, University of Sao Paulo, São Paulo, Brazil

^5^Department of Clinical Translation, Max Planck Institute of Psychiatry, Munich, Germany

^6^German Center for Mental Health (DZPG), partner site Munich/Augsburg, Munich, Germany

^#^Shared co-first authors

***Correspondence:**

Moritz J. Rossner

E-mail: [moritz.rossner@med.uni-muenchen.de](mailto:moritz.rossner@med.uni-muenchen.de)

**Keywords: oligodendrocytes, myelination, Tcf4, Olig2, mouse behaviors, mental disorders**

# Supplementary materials and methods

**Behavioral tests**

Based on the Research Domain Criteria (RDoC) concept (Simmons and Quinn, 2014), all the conducted behavioral tests were categorized to respective domains as described previously (Volkmann et al., 2021; Stephan et al., 2022): Rotations, distance/time/entries in the center, and fecal pellets in the open field test, and the immobile time in the tail suspension test were mapped to the negative valence domain due to their involvement of responses to aversive situations or context, such as anxiety. The sucrose preference and the place learning in the IntelliCage were mapped to the positive valence domain as they are the responses to positively motivational situations, such as rewarding seeking and habit learning. The spontaneous alternations in the Y maze test, the serial reversal learning in the IntelliCage, freezing behaviors in the fear conditioning and remote fear test were mapped to the cognitive domain as these are behaviors associated with cognitive functions. Social preference, discrimination index, and the free interaction in the social interaction test were mapped to the social domain. The mean traveling speed in the open field test, the general activity and nocturnality in the IntelliCage were mapped to the arousal domain as this system links to energy balance and sleep, such as locomotor activity and circadian rhythms. Finally, the prepulse inhibition was mapped to the sensorimotor gating domain for the control, execution, and refinement of motor behaviors.

*Open field test*

Mice were placed in an open field (OF) arena (50 cm x 50 cm x 50 cm) to examine their spontaneous and novelty-induced exploratory behaviors for 10 min, and the video recording started automatically once the experimenter left the recording area. The center region was defined by excluding a 5 cm-wide border along the walls and the 10 cm x 10 cm square in each corner.

*Y maze test*

The Y maze consists of three identical arms (A, B, and C). Mice were placed in arm A, and the video recording started automatically once the experimenter left the recording area. The working memory capacity was evaluated by measuring spontaneous alternations in the Y maze for 10 min. Spontaneous alternations were determined by the frequency of complete sequences of visits (choices) to each arm without repetition (e.g., A-B-C, B-A-C, or B-C-A). The percentage of alternations was calculated by:

$$Spontaneous alternations= \frac{\sum(full sequences)}{choices-2} \times100\%$$

*IntelliCage system*

The IntelliCage system (TSE Systems, Berlin, Germany) monitors home cage behaviors and facilitates learning paradigms. The IntelliCage consists of four corners, each of which has two doors with access to water bottles when the door is opened by predefined actions (e.g. nosepoke, visit to the assigned corner) of mice. The registration of a known RFID to a corner was counted as a visit, and visits to a corner, nosepokes and licks at a water bottle were recorded continuously throughout all paradigms. Food was provided *ad libitum*. The paradigms were performed as follows:

1. 48-hr free adaptation: doors opened all the time, and mice had free access to water bottles in all corners.
2. 48-hr free adaptation with doors open upon visit: doors opened when a mouse entered a corner, and mice could visit all corners.
3. 72-hr nosepoke adaptation: the door opened and the mouse had access to the water bottle for 7 seconds (sec) when it entered a corner and performed one or more nosepokes at the door.
4. 48-hr place learning: each mouse was assigned a specific corner and could access the water bottles only after making a nosepoke in its assigned corner.
5. 6-day serial reversal learning: place learning protocol repeated with a new assigned drinking corner for each mouse every 24 hr.
6. 24-hr sucrose preference: doors opened all the time, and mice had free access to all corners; each corner contained one bottle with 4% sucrose solution and the other with regular drinking water.

The home cage activity and the nocturnality were monitored continuously over the first 7 days. Place preference, sucrose preference, and nocturnality were determined by the preference score:

$$Preference score= \frac{A-B}{A+B}$$

*A* represents the number of correct trials, defined as visits to the assigned corner with at least one nosepoke, licks at the sucrose solution bottle, or nighttime visits. *B* represents the number of incorrect trials, defined as nosepokes in non-assigned corners, licks at the water bottle, or daytime visits.

In serial reversal learning, the percentages of correct trials over total trials were quantified each day and plotted throughout 6 days as a connected scatterplot, and the area under the curve (AUC) of each group was calculated for better comprehension of the learning ability.

*Social interaction test*

Because males tend to be aggressive to male conspecifics and exhibit sexual behaviors toward females, oophorectomized female mice were used as the social stimulus to reduce variability arising from the sexual interaction rather than regular social interaction. Ovariectomy can robustly deplete the endogenous sex hormones, rendering the oophorectomized mice “sex-neutral” (Rowe et al., 2023). This allows the same behavioral dimensions to be measured in both sexes without sexually, territorially or aggressively influenced behavior. Furthermore, 129/Sv were used as stimulus animals due to their different skin color for better detection and differentiation among mice in the following video analysis.

The social interaction test was conducted for 3 consecutive days to examine the social preference and the social memory in mice.

Day 1: Habituation

Mice were placed in an OF arena (50 cm x 50 cm x 50 cm) with two empty wire cups positioned on a blue or green coaster for spatial orientation. The same color of coasters remained in fixed positions at opposite corners of the arena throughout the test. Mice were allowed to explore the arena for 10 min.

Day 2: Social interaction testing

The test consisted of three stages:

1. Pre-test: mice were placed in an OF arena with two wire cups, each containing a ball of crumpled paper, on a blue or green coaster for 10 min.

2. Social preference test: a stimulus mouse as a social stimulus was placed in a wire cup, and a wooden cube as a non-social stimulus was put in the other cup. Mice were allowed to explore for 10 min, and their preference for a social over a non-social stimulus was assessed. The social preference index was calculated by:

$$Social preference index= \frac{{Time}_{social}- {Time}_{Nonsocial}}{{Time}_{social}+ {Time}_{Nonsocial}}$$

3. Social memory test: the same stimulus mouse from the previous stage was placed in the same wire cup, and a novel stimulus mouse was placed in the other. Mice were allowed to explore for 10 min, and their ability to differentiate the novel and the familiar mice was assessed. The social discrimination index was calculated by:

$$Social discrimination index= \frac{{Time}_{Novel}- {Time}_{Familiar}}{{Time}_{Novel}+ {Time}_{Familiar}}$$

Day 3: Free interaction testing

Mice were placed in an OF arena and allowed to freely interact with a novel stimulus mouse for 10 min. The distance between the two mice’ heads, reflecting the interaction level, were analyzed using DeepLabCut (Lauer et al., 2022).

*Prepulse inhibition (PPI) test*

Mice were habituated to the enclosures with white noise in the background for 10 min for 2 consecutive days. The PPI test was conducted on the third day to assess the sensorimotor gating function. Startle responses were measured automatically via movement-induced vibrations of the base plate in the startle-response enclosures (SR-LAB, San Diego Instruments, San Diego, CA, USA). Background noise was maintained at 65 dB. For short-term habituation, a 40-milliseconds (ms), 115 dB pulse was presented 10 times prior to the test sequence. PPI was assessed using a non-startling 20 ms prepulse at 70, 75, or 80 dB, followed 100 ms later by a 115 dB pulse. Baseline startle response was measured by presenting the 115 dB pulse alone. Each condition was repeated across 10 trials in a pseudorandomized order. The inter-trial intervals ranged from 8 to 22 sec. The percentage of PPI was calculated by:

$$\%PPI= \frac{Pulse response-Prepulse response}{Pulse response} \times100$$

*Tail suspension test*

The tail suspension test was used to examine behavioral despair or depression-like behavior in mice (Cryan et al., 2005). Mice were suspended by the tail using adhesive tape attached to a horizontal bar positioned 30 cm above the surface for 6 min. Immobility was measured by the duration during which no movements of the entire mouse body were recorded.

*Fear conditioning and remote fear memory test*

The fear conditioning test was conducted in soundproof boxes equipped with dim lighting and a speaker (Ugo Basile, Siena, Italy) for 3 consecutive days.

Day 1: Conditioning

An enclosure, with a grid floor capable of delivering electrical foot shocks and walls covered by black and white striped paper for visual context, was placed in each box. Mice were put into the enclosure for 4 min to undergo fear conditioning. In the first 2 min, the baseline freezing responses in a novel environment were recorded. Next, an auditory cue was presented for 20 sec, followed by a 2-sec foot shock of 0.6 mA. After a 30-sec break, the cue was presented again for 20 sec, followed by another 2-sec foot shock.

Day 2: Contextual memory

Mice were placed in the enclosure for 2 min without any cues or foot shocks. The freezing behavior in response to the context was recorded and analyzed.

Day 3: Cue memory

A transparent plastic cylinder (25 cm in diameter) was placed above a gray floor in the box to prevent context-triggered responses. Mice were placed into the cylinder for 4 min. After habituation for 2 min, the auditory cue was presented for 2 min to assess their freezing behavior in response to the cue. The cylinder and the gray floor were cleaned using only sodium dodecyl sulfate, but not ethanol, to avoid contextual responses.

About 20 days after the fear conditioning test, the remote fear memory test was performed to examine the long-term fear memory in the mice. The test was conducted for 2 consecutive days with similar procedures to the fear conditioning test.

Day 1: Contextual memory

Mice were put into the enclosure with black and white striped walls for 2 min without any cues or foot shocks. The freezing behavior in response to the context was recorded and analyzed.

Day 2: Cue memory

Mice were placed in the transparent plastic above the gray floor in the box for 4 min. After habituation for 2 min, the auditory cue was presented for 2 min to assess their freezing behavior in response to the cue.

# Supplementary figures

**Figure S1. *Tcf4*-*Olig2* dHet mice showed no alterations in the social valence system.**

**(A)** The social preference index, and **(B)** the social discrimination index in the social interaction test (SI). Two-way ANOVA test. G, genotype effect; S, sex effect; G x S, genotype x sex interaction. Data = Mean ± SD. WT: n = 33 (16 males and 17 females), dHet: n = 27 (14 males and 13 females).

**Figure S2. Significant effect of sex on serial reversal learning in the cognitive valence systems.**

**(A)** The percentage of spontaneous alterations in the Y maze test. The learning curves of **(B)** male and **(C)** female animals in the serial reversal learning paradigm in the IntelliCage. **(C)** The area under the curve (AUC) of the learning curve of serial reversal learning paradigm. Two-way ANOVA test: ***, *p* < 0.001. G, genotype effect; S, sex effect; G x S, genotype x sex interaction. Data = Mean ± SD. WT: n = 33 (16 males and 17 females), dHet: n = 27 (14 males and 13 females).

**Figure S3. Significant effect of sex on nocturnality in the arousal valence system.**

**(A)** The mean traveling speed in the OF test. **(B)** The general activity and **(C)** the nocturnality in the IntelliCage. Two-way ANOVA test: ***, *p* < 0.001. G, genotype effect; S, sex effect; G x S, genotype x sex interaction. Data = Mean ± SD. WT: n = 33 (16 males and 17 females), dHet: n = 27 (14 males and 13 females).

# References

Cryan, J. F., Mombereau, C., and Vassout, A. (2005). The tail suspension test as a model for assessing antidepressant activity: Review of pharmacological and genetic studies in mice. *Neuroscience & Biobehavioral Reviews* 29, 571–625. doi: 10.1016/j.neubiorev.2005.03.009

Lauer, J., Zhou, M., Ye, S., Menegas, W., Schneider, S., Nath, T., et al. (2022). Multi-animal pose estimation, identification and tracking with DeepLabCut. *Nat Methods* 19, 496–504. doi: 10.1038/s41592-022-01443-0

Rowe, A. A., Issioui, Y., Johnny, B., and Wert, K. J. (2023). Murine Orchiectomy and Ovariectomy to Reduce Sex Hormone Production. *J Vis Exp*. doi: 10.3791/64379

Simmons, J. M., and Quinn, K. J. (2014). The NIMH Research Domain Criteria (RDoC) Project: implications for genetics research. *Mamm Genome* 25, 23–31. doi: 10.1007/s00335-013-9476-9

Stephan, M., Schoeller, J., Raabe, F. J., Schmitt, A., Hasan, A., Falkai, P., et al. (2022). Spironolactone alleviates schizophrenia-related reversal learning in Tcf4 transgenic mice subjected to social defeat. *Schizophr* 8, 77. doi: 10.1038/s41537-022-00290-4

Volkmann, P., Stephan, M., Krackow, S., Jensen, N., and Rossner, M. J. (2021). PsyCoP – A Platform for Systematic Semi-Automated Behavioral and Cognitive Profiling Reveals Gene and Environment Dependent Impairments of Tcf4 Transgenic Mice Subjected to Social Defeat. *Front. Behav. Neurosci.* 14, 618180. doi: 10.3389/fnbeh.2020.618180
